# Sirt1 regulates microglial activation and inflammation following oxygen-glucose deprivation/reoxygenation injury by targeting the Shh/Gli-1 signaling pathway

**DOI:** 10.1007/s11033-022-08167-6

**Published:** 2023-02-01

**Authors:** Hongyan Liao, Jiagui Huang, Jie Liu, Huimin Zhu, Yue Chen, Xuemei Li, Jun Wen, Qin Yang

**Affiliations:** Department of Neurology, the First Affiliated Hospital of Chongqing Medical University, 1 Youyi Road, Yuzhong District, Chongqing, 400016 China

**Keywords:** Microglial activation, Neuroinflammation, Oxygen-glucose deprivation/reoxygenation, Sirtuin 1, Sonic hedgehog signaling pathway

## Abstract

**Background:**

Cerebral ischemic injury leads to over-activation of microglia, which release pro-inflammatory factors that deteriorate neurological function during the acute phase of stroke. Thus, inhibiting microglial over-activation is crucial for reducing ischemic injury. Sirtuin 1 (Sirt1) has been shown to play a critical role in stroke, neurodegenerative diseases and aging. However, the effect of Sirt1 on the regulation of microglial activation following cerebral ischemic injury, as well as the underlying mechanism, remain unknown. Therefore, the purpose of the present study is to mainly investigate the effect of Sirt1 on oxygen-glucose deprivation/reoxygenation (OGD/R)-treated N9 microglia following treatment with the Sirt1 agonists resveratrol and SRT1720 and the Sirt1 antagonist sirtinol.

**Methods:**

Cell viability, Apoptosis, activation and inflammatory responses of microglia, expressions and activity of Shh signaling pathway proteins were detected by Cell Counting Kit 8, Flow Cytometry, immunocytochemistry, ELISA, and Western blotting, respectively.

**Results:**

The results demonstrated that treatment with resveratrol or SRT1720 could inhibit the activation of microglia and inflammation during OGD/R. Moreover, these treatments also led to the translocation of the GLI family zinc finger-1 (Gli-1) protein from the cytoplasm to the nucleus and upregulated the expression of Sonic hedgehog (Shh), Patched homolog-1 (Ptc-1), smoothened frizzled class receptor and Gli-1. By contrast, the inhibition of Sirt1 using sirtinol had the opposite effect.

**Conclusion:**

These findings suggested that Sirt1 may regulate microglial activation and inflammation by targeting the Shh/Gli-1 signaling pathway following OGD/R injury.

**Graphical abstract:**

**Figure:**
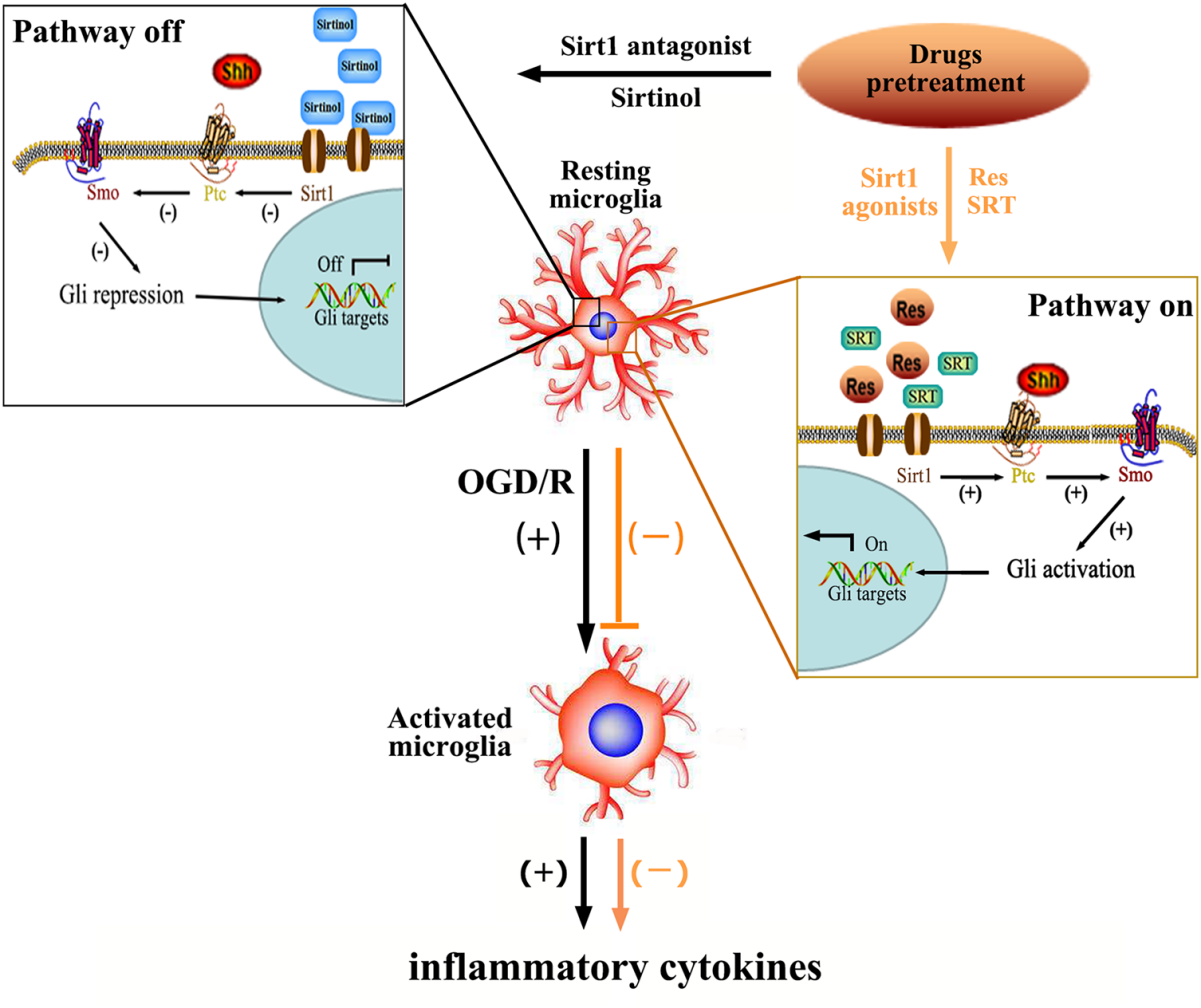
Schematic representation of Sirt1 regulating the microglial activation and inflammation following oxygen-glucose deprivation/reoxygenation injury via mediation of Shh/Gli-1 signaling pathway.

**Supplementary Information:**

The online version contains supplementary material available at 10.1007/s11033-022-08167-6.

## Introduction

Microglia are immune cells residing in the brain and spinal cord that can modulate local inflammatory and immune responses [1, 2]. Ischemic injury can activate microglia, which act as a double-edged sword with respect to stroke [3]. On the one hand, microglial activation promotes the release of pro-inflammatory cytokines, such as IFN-γ, IL-6, TNF-α and IL-1β, and aggravates ischemic injury. On the other hand, it also causes the release of anti-inflammatory cytokines, such as IL-10, and improves neurological function [1–4]. Therefore, regulating microglial activation is important in order to reduce injury and promote functional recovery after stroke.

Silent information regulator factor 2-related enzyme 1 (sirtuin 1/Sirt1), a nicotinamide adenine dinucleotide-dependent protein deacetylase, can modulate a wide range of biological processes, including oxidative stress, inflammation, aging, apoptosis and autophagy [5]. Previous studies have demonstrated that Sirt1 can exert protective effects against cerebral ischemia, Alzheimer’s disease, traumatic brain injury and Parkinson’s disease [6–10]. In addition, Sirt1 can regulate the activation of microglia [11–13]. However, the molecular mechanism underlying the effect of Sirt1 on the regulation of microglial activation has not been fully elucidated.

The Sonic hedgehog (Shh) signaling pathway has a key role in stem cell behavior, organ differentiation, embryonic development, axonal growth and regulation in the developing as well as the adult brain [14–16]. Following ischemic injury, neurodegeneration, infection or trauma in the brain or spinal cord, Shh signaling is activated and can improve neurological function by regulating inflammation, oxidative stress and apoptosis [15, 17–22]. For example, the BCL6/BCOR/SIRT1 complex has been reported to promote neurogenesis and suppress medulloblastoma formation by inhibiting Shh signaling [23]. Moreover, Sirt1 can regulate the viability of NIH3T3 cells and neurons following OGD/R injury in vitro via Shh signaling [13, 24]. However, whether Sirt1 can regulate microglial activation after stroke and whether this would involve the Shh signaling pathway remains not quite clear.

Therefore, in the present study, cultured microglia were exposed to OGD/R to mimic ischemia/reperfusion injury *in vitro and* were used to determine whether Sirt1 could regulate microglial activation and inflammation following OGD/R injury in vitro and whether these effects were mediated via Shh/GLI family zinc finger-1 (Gli-1) signaling. The effects of Sirt1 agonists and antagonists on microglial viability, activation and inflammation, as well as the nuclear translocation of Gli-1 and the expression of Shh signaling pathway components following OGD/R injury were also examined.

## Materials and methods

### Culture of N9 microglia

N9 microglia (kindly provided by the Department of Anesthesia, The Affiliated Children’s Hospital of Chongqing Medical University, Chongqing, China) were cultured in DMEM/F-12 (HyClone; Cytiva) supplemented with 10% FBS (PAN-Biotech), 100 U/ml streptomycin and 100 U/ml penicillin at 37 °C in a humidified atmosphere with 5% CO_2_. The cells were digested with 0.125% trypsin and subcultured at a 1:3 ratio when they reached 80% confluence.

### OGD/R model

The OGD/R model was established in N9 microglia according to previously described methods [13].For oxygen-glucose deprivation, microglia were maintained in D-Hanks solution with a humidified anaerobic incubator (Thermo 3111; Thermo Fisher Scientific, Inc.) containing 94% N_2_, 1% O_2_ and 5% CO_2_ at 37 °C for 150 min. For reoxygenation, microglia were incubated in a humidified normoxic environment with complete medium for 24 h.

### Drug treatment

To determine whether Sirt1 was associated with Shh signaling, microglia were divided into five groups as follows: (i) Normal (Nor) group, microglia (~ 100,000 cells/ml) cultured in complete medium without OGD/R; (ii) control (Ctrl) group, microglia (~ 100,000 cells/ml) cultured in complete medium containing 0.4% DMSO for 24 h, then subjected to OGD/R as aforementioned; (iii) resveratrol (Res) group, microglia (~ 100,000 cells/ml) cultured in complete medium containing the Sirt1 agonist resveratrol (20 µmol/l; 99% purity; MilliporeSigma) for 24 h, then subjected to OGD/R as aforementioned; (iv) SRT1720 (SRT) group, microglia (~ 100,000 cells/ml) cultured in complete medium containing the Sirt1 agonist SRT1720 hydrochloride (0.5 µmol/l; 99.92% purity; MedChemExpress) for 24 h, then subjected to OGD/R as aforementioned; and (v) sirtinol group, microglia (~ 100,000 cells/ml) cultured in complete medium containing the Sirt1 antagonist sirtinol (10 µmol/l; 98% purity; MedChemExpress) for 24 h, then subjected to OGD/R as aforementioned. All drugs were dissolved in DMSO (cat. no. D2650; MilliporeSigma) respectively and then diluted to the corresponding concentration with culture medium.

### Cell counting Kit-8 (CCK-8) assay

Microglial viability was estimated using a CCK-8 assay (Dojindo Laboratories, Inc.). Briefly, N9 microglia were seeded in poly-L-lysine-coated 96-well plates (~ 5,000 cells/well) and received the aforementioned treatments. The cells were incubated for 4 h at 37 °C, and the CCK-8 solution was then added to each well (10 µl in 100 µl culture). Absorbance was measured at 450 nm using a microplate reader (Thermo Labsystems).

### Flow cytometry

The N9 microglia were collected after treatment with each drug following OGD/R injury, washed three times in ice-cold PBS and resuspended in PBS at a concentration of 1 × 10^6^ cells/ml; subsequently, 5 µl Annexin V fluorescein isothiocyanate and propidium iodide were added, followed by culture at room temperature in the dark for 15 min; next detected the apoptotic cells by flow cytometry (FACSCalibur™; BD Biosciences). Then the data were analyzed with CytExpert 2.0 software (Beckman Coulter) which the percentage of early apoptosis and late apoptosis were showed in the upper right quadrant and the lower right quadrant respectively. Finally, the percentage of apoptosis of microglia can be quantified as the sum of the percentage of early apoptosis and late apoptosis.

### ELISA

The levels of the inflammatory cytokine TNF-α, IL-1β and IL-10 in cell supernatants were measured using ELISA kits (cat. nos. EMC102a, EMC001b and EMC005, respectively; Neobioscience Technology Co., Ltd.) according to the manufacturer’s instructions.

### Immunocytochemistry

Microglia from each group were seeded on poly-L-lysine-coated coverslips (500 cells/coverslip), fixed with 4% formaldehyde solution at room temperature for 30 min, incubated with 1% Triton X-100 at 37 °C for 30 min, subsequently blocked with 5% goat or donkey serum (cat. no. SAP-9100; Beijing Zhongshan Golden Bridge; cat. no. D9663; MilliporeSigma) at 37 °C for 1 h. The cells were then incubated overnight at 4 °C with anti-ionized calcium-binding adaptor molecule 1 (Iba1; monoclonal; rabbit; 1:100 dilution; cat. no. ab178847; Abcam) or anti- Gli-1 (polyclonal; rabbit; 1:100; cat. no. ab273018; Abcam) antibody. While there were no primary antibodies in the negative controls. The cells were incubated with Cy3-conjugated goat anti-rabbit IgG (1:200; cat. no. SA00009-2; ProteinTech Group, Inc.) at 37 °C for 1 h the next day. The nuclei were counterstained with DAPI (cat. no. C1005; Beyotime Institute of Biotechnology) in the dark at 25 °C for 5 min. All images were captured using an A1 + R laser confocal microscope (Nikon Corporation).

### Western blotting

Microglia were homogenized in RIPA lysis buffer (cat. no. P0013C; Beyotime Institute of Biotechnology) containing 1% phenylmethane sulfonyl fluoride (cat. no. ST506; Beyotime Institute of Biotechnology) and incubated on ice for 30 min. The protein concentration of each extract was measured using a BCA Protein Assay Reagent Kit (cat. no. P0010; Beyotime Institute of Biotechnology) following centrifugation (13,684 x g at 4 °C) for 10 min. A total of 40 µg protein per lane was loaded and separated via 10% SDS-PAGE, then transferred onto PVDF membranes (cat. no. 03010040001; MilliporeSigma). The membranes were blocked with 5% skimmed milk at room temperature for 2 h, then incubated overnight at 4 °C with the following primary antibodies: Monoclonal rabbit anti-Iba1 antibody (1:1000 dilution; cat. no. ab178847; Abcam); polyclonal rabbit antibodies against Shh, Patched homolog-1 (Ptc-1), smoothened frizzled class receptor (Smo), Gli-1, caspase-3, Bax (1:1000; cat. nos. ab19897, ab53715, ab235183, ab273018, ab13847, ab263897; all from Abcam), IL-10, TNF-α and IL-1β (1:1,000; cat. nos. bs-0698R, bs-0078R and bs-0812R; all from BIOSS); and monoclonal rabbit anti-GAPDH (loading control; 1:1000; cat. no. AF1186; Beyotime Institute of Biotechnology). The membranes were incubated with horseradish peroxidase-conjugated AffiniPure goat anti-rabbit antibody (1:2000; cat. no. ZB-2301; Beijing Zhongshan Golden Bridge) at 37 °C for 1 h the next day. Finally, the protein bands were visualized using enhanced chemiluminescence reagent (cat. no. P0018FS; Beyotime Institute of Biotechnology), and their intensities were analyzed using Quantity One v. 4.6.6 software (Bio-Rad Laboratories, Inc.).

### Statistical analysis

The data are expressed as the mean ± standard deviation. Statistical analysis was carried out using one-way ANOVA analysis of variance followed by Tukey’s post-hoc test with SPSS 22.0 (IBM Corp.). P < 0.05 was considered as a statistically significant difference.

## Results

### Effects of Sirt1 on the viability and apoptosis of microglia following OGD/R injury in vitro

Firstly, we consulted the relevant literature to determine the dosages of these drugs [13, 24–28]. Then our team has studied dosages of resveratrol and SRT1720 following OGD/R injury in the pre experiment stage by CCK-8 assays and the results showed that the optimal drug concentrations of resveratrol and SRT1720 were 20 µmol/l and 0.5 µmol/l respectively (P < 0.05, Fig. S1A,B). However, in the previous articles published by our team [13, 24], it has been studied that the drug concentration of sirtinol following OGD/R injury was 10 µmol/l, so the present study is directly discussed on the previous research.

**Fig. 1:**
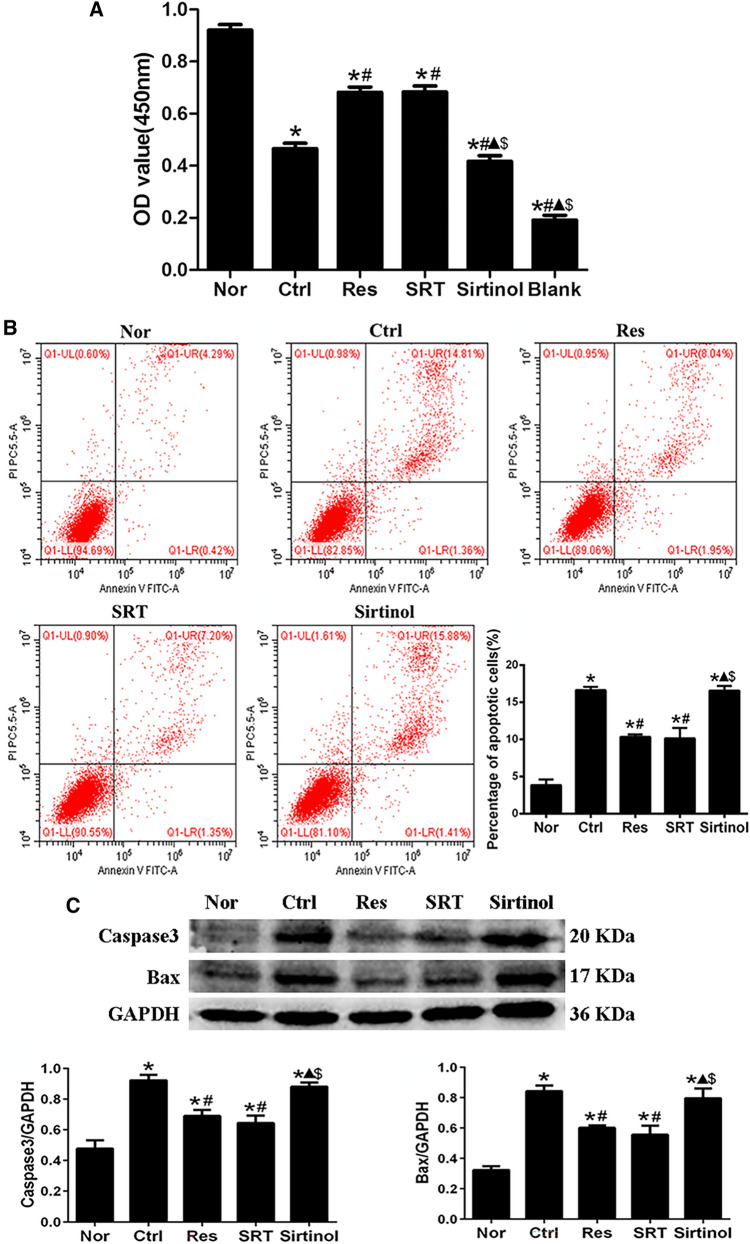
Effects of Sirt1 on the viability and apoptosis of microglia following OGD/R injury in vitro **A** Effect of Sirt1 on the viability of microglia following OGD/R injury. Compared with the Ctrl group, Res and SRT treatment significantly increased while sirtinol significantly reduced the viability of microglia. **B** Effect of Sirt1 on the apoptosis of microglia following OGD/R injury, as determined using flow cytometry. **C** Effect of Sirt1 on the expression of apoptosis proteins in microglia following OGD/R injury, as determined using western blotting. ^*^P < 0.05 vs. Nor; ^#^P < 0.05 vs. Ctrl; ^▲^P < 0.05 vs. Res; ^$^P < 0.05 vs. SRT; ANOVA. n = 3

The effects of Sirt1 on microglial viability were examined using CCK-8 assays following OGD/R injury. Compared with the Nor group, the viability of microglia in the Ctrl, Res, SRT and sirtinol groups was significantly decreased (P < 0.05, Fig. 1 A). Res and SRT treatments had similar effects on the viability of microglia relative to the Ctrl group (Fig. 1 A). However, viability was significantly decreased in the sirtinol group compared with the Res and SRT groups (P < 0.05, Fig. 1 A).

Microglial apoptosis, as well as the protein expression levels of caspase-3 and Bax, were examined using flow cytometry and western blot analysis, respectively. The percentage of apoptotic microglia and the protein expression levels of caspase-3 and Bax were significantly increased in the Ctrl, Res, SRT and sirtinol groups compared with the Nor group (P < 0.05). However, these were significantly decreased in the Res and SRT groups compared with the Ctrl group (P < 0.05, Fig. 1B and C), and significantly increased in the sirtinol group compared with the Res and SRT groups following OGD/R injury (P < 0.05, Fig. 1B and C). These results demonstrated that Sirt1 could regulate the viability and apoptosis of microglia following OGD/R injury in vitro.

### Effects of Sirt1 on activation of microglia and inflammation following OGD/R injury in vitro

**Fig. 2:**
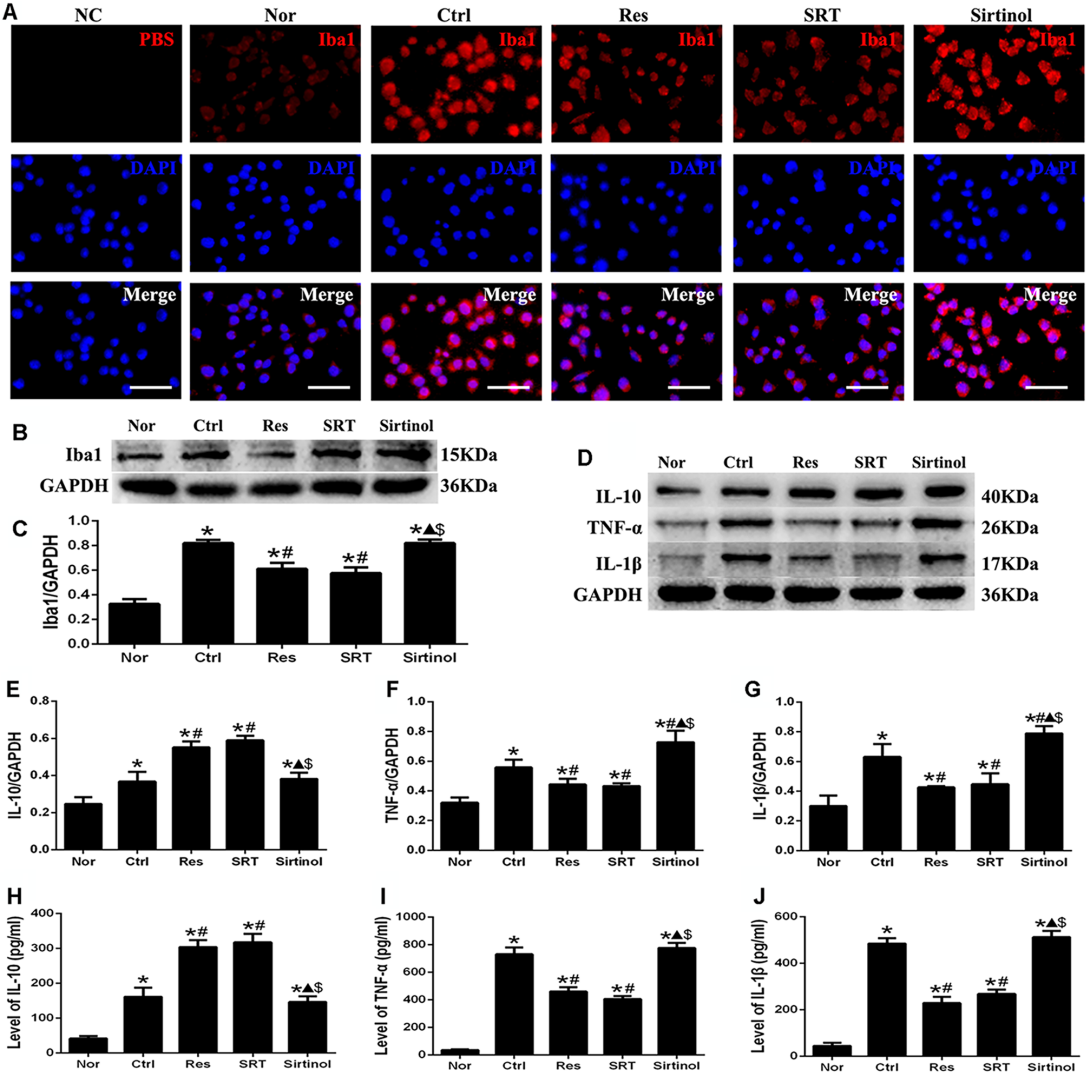
Effects of Sirt1 on microglial activation and inflammation following OGD/R injury in vitro(**A**) Immunofluorescence images showed the activation of microglia in each group following OGD/R injury. The anti-Iba1 antibody was replaced with PBS to serve as NC. Red, Iba1; blue, DAPI. Scale bar, 50 μm. (**B** and **C**) Effect of Sirt1 on the protein expression of Iba1, as evaluated using western blotting. (**D**-**J**) Effect of Sirt1 on the secretion of the inflammatory cytokines IL-10, TNF-α and IL-1β following OGD/R injury, as assessed using ELISA and western blotting. ^*^P < 0.05 vs. Nor; ^#^P < 0.05 vs. Ctrl; ^▲^P < 0.05 vs. Res; ^$^P < 0.05 vs. SRT; ANOVA. n = 3

Morphological changes and upregulation of Iba1 protein expression levels are characteristic of microglial activation [4]. Therefore, morphological changes and Iba1 protein expression levels were examined using immunofluorescence and western blotting. In the Nor group, almost all microglia exhibited small cell bodies, thin-branched processes and weak fluorescence. However, activated microglia, which were characterized by enlarged cell bodies, thick processes and stronger fluorescence intensity, were observed in the Ctrl and sirtinol groups following OGD/R injury. Moreover, Res and SRT reversed the signs of microglial activation compared with the Ctrl and sirtinol groups following OGD/R injury (P < 0.05, Fig. 2 A). In addition, the protein expression of Iba1 exhibited the same trend, as determined by fluorescence intensity (P < 0.05, Fig. 2B and C).

Activated microglia serve a dual role during ischemia, releasing pro-inflammatory cytokines (such as TNF-α and IL-1β) that can aggravate ischemic injury, whilst promoting the release of anti-inflammatory cytokines (such as IL-10) to reduce ischemic injury [2]. Therefore, ELISA and western blotting were used to examine the protein levels of TNF-α, IL-1β and IL-10 in culture supernatants and in cell lysates, respectively, following OGD/R. The protein levels of TNF-α, IL-1β and IL-10 were significantly increased in the culture supernatant and in the cell lysates 24 h after OGD/R injury in the Ctrl, Res and SRT and sirtinol groups compared with those in the Nor group (P < 0.05, Fig. 2D-J). In the Res and SRT groups, the protein levels of TNF-α and IL-1β were significantly reduced and those of IL-10 were significantly increased compared with the Ctrl group (P < 0.05, Fig. 2D-J). In the sirtinol group, the protein levels of TNF-α and IL-1β were significantly increased and those of IL-10 were significantly decreased compared with the Res and SRT groups (P < 0.05, Fig. 2D-J). These results suggested that Sirt1 could regulate activation of N9 microglia and inflammation following OGD/R injury *in vitro.*

### Effects of Sirt1 on nuclear translocation of Gli-1 and expression of shh, Ptc-1, smo and Gli-1 in N9 microglia following OGD/R injury in vitro

Shh signaling plays an important role in embryogenesis, cell proliferation and differentiation, as well as tissue repair [16]. Therefore, the subsequent experiments were used to determine whether Sirt1 affected the nuclear translocation of Gli-1 and the expression levels of components of the Shh signaling pathway, such as Shh, Ptc-1, Smo and Gli-1.

**Fig. 3:**
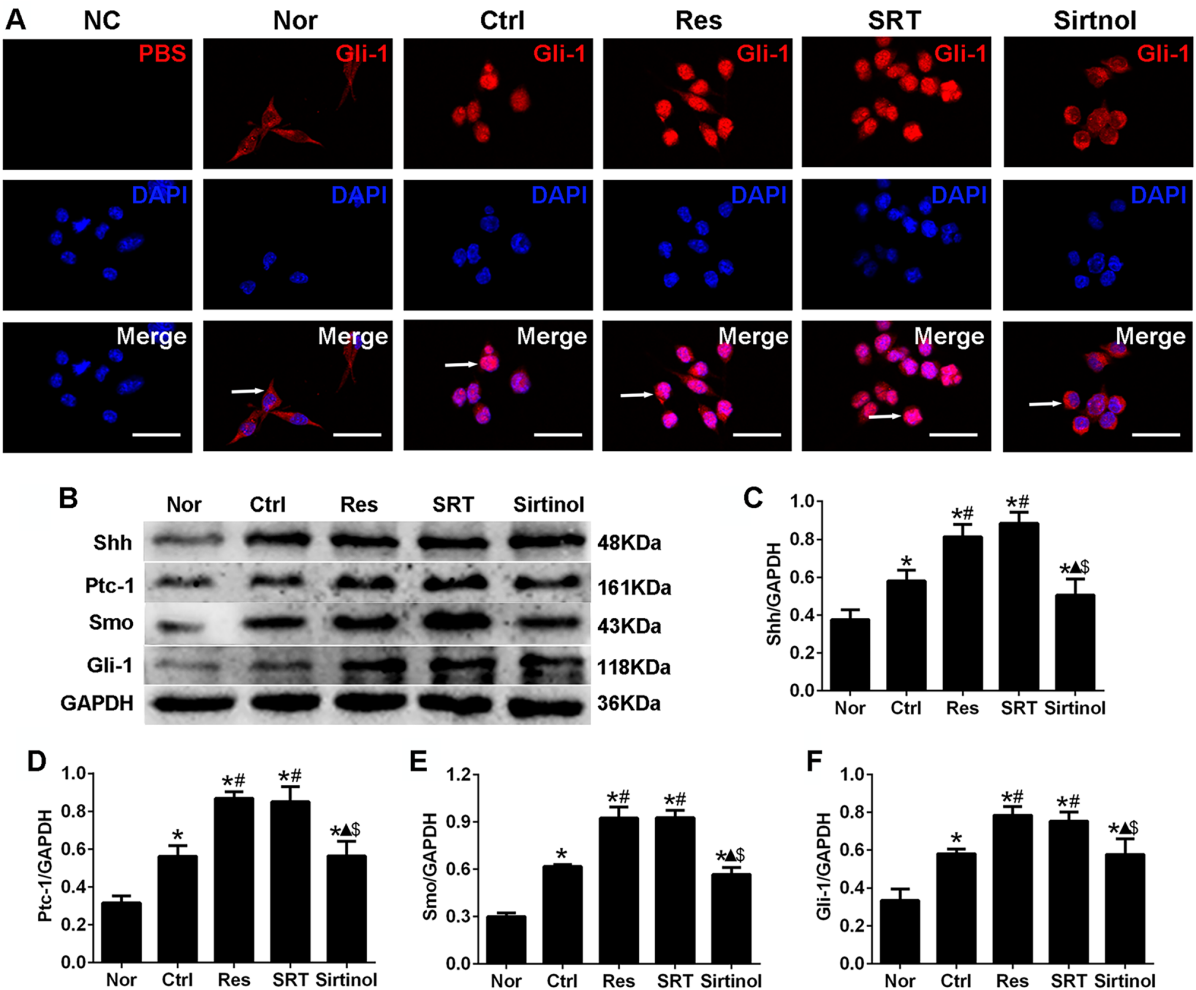
Effects of Sirt1 on the translocation of Gli-1 molecules and the expression of Shh, Ptc-1, Smo and Gli-1 in N9 microglia following OGD/R injury in vitro (**A**) Effects of Sirt1 on the translocation of Gli-1 following OGD/R injury. Gli-1 accumulated in the cytoplasm in the Nor group, but it was partly transferred to the nucleus in the Ctrl and sirtinol groups and almost completely transferred to the nucleus in the Res and SRT groups. The anti-Gli-1 antibody was replaced with PBS to serve as NC. Red, Gli-1; blue, DAPI. Scale bar, 50 μm. (**B**-**F**) Effect of Sirt1 on Shh, Ptc-1, Smo and Gli-1 protein expression in microglia following OGD/R injury, as assessed using western blotting. ^*^P < 0.05 vs. Nor; ^#^P < 0.05 vs. Ctrl; ^▲^P < 0.05 vs. Res; ^$^P < 0.05 vs. SRT; ANOVA. n = 3

Immunofluorescence staining demonstrated that Gli-1 accumulated in the cytoplasm in the Nor group and was also mostly observed in the cytoplasm in the Ctrl group. In the Res and SRT groups, Gli-1 was translocated from the cytoplasm to the nucleus. However, in the sirtinol group, Gli-1 was mostly observed in the cytoplasm (Fig. 3 A). Moreover, western blot analysis indicated that the protein expression levels of Shh, Ptc-1, Smo and Gli-1 were significantly increased in the Res and SRT groups compared with the Ctrl group and were significantly decreased in the sirtinol group compared with the Res and SRT groups (P < 0.05, Fig. 3B-F). These results indicated that Sirt1 regulated the nuclear translocation of Gli-1 and the expression levels of components of the Shh signaling pathway in N9 microglia following OGD/R injury *in vitro.*

## Discussion

The present study demonstrated that stimulation with the Sirt1 agonists Res or SRT could suppress the microglial activation and apoptosis, reduce inflammation, trigger Gli-1 translocation from the cytoplasm to the nucleus and upregulate the protein expression levels of Shh, Ptc-1, Smo and Gli-1 following OGD/R in vitro. By contrast, inhibition of Sirt1 using sirtinol had the opposite effect. These findings suggest that, following OGD/R injury, Sirt1 regulates microglial activation and inflammation via the Shh/Gli-1 signaling pathway.

Activated microglia acquire either an ‘M1’ phenotype and release pro-inflammatory cytokines, or an ‘M2’ phenotype associated with the release of anti-inflammatory cytokines [2]. Butturini et al. [29] reported that hypoxia induced the phosphorylation and S-glutathionylation of STAT1, leading to aberrant M1 microglial activation. In an in vitro lipopolysaccharide-induced microglial activation model, sevoflurane reduced microglial activation by suppressing NF-κB and MAPK signaling [30]. Stromal cell-derived factor-1 can induce microglial activation through the PI3K and ERK signaling pathways [31]. Salidroside suppresses M1 microglial polarization and promotes spinal cord injury functional recovery through AMPK/mTOR-mediated autophagic flux stimulation [32]. Res can suppress M1 microglial polarization via peroxisome proliferator-activated receptor γ coactivator 1-α or Sirt1 [33]. Melatonin was also shown to inhibit microglial activation via the SIRT1/nuclear factor erythroid 2-related factor 2 pathway [34]. The present study demonstrated that stimulation with the Sirt1 agonists, Res or SRT, could inhibit activation of microglia by triggering the translocation of Gli-1 molecules and upregulating the expression of components of the Shh signaling pathway. Sirt1 inhibition using sirtinol had the opposite effect. Taken together, these findings suggest that microglial activation may be regulated through various signaling pathways and could be targeted using multiple drugs.

Lu et al. [27] reported that activated Sirt1 can inhibit microglial activation by inhibiting Wnt/β-catenin signaling. In a mouse model of type-2 diabetes, Sirt1 activation inhibited microglial activation by upregulating BDNF [35]. Moreover, Sirt1 can reduce microglia-dependent amyloid-β toxicity by inhibiting NF-κB signaling [36]. These findings suggested that Sirt1 may affect microglial activation through various signaling pathways. In addition, according to the literature, we know that resveratrol can activate microglia, promote the M2 polarization of microglia and reduce neuroinflammation [37, 38]. Duan et al. [39] reported that SRT2140, an Sirt1 agonist, could attenuate CUMS-induced depressive-like behaviors via shifting microglial polarization toward the M2 phenotype. SRT1720, an agonist of Sirt1, can improve cell viability, promote M2 microglia polarization, suppress inflammatory response and inhibit oxidative damage via modulation of ROS-mediated NLRP3 inflammasome signaling after subarachnoid hemorrhage [40]. At present, our team has not yet conducted research on the polarization of microglia, which will be the next research goal of our team to explore the pathway how to regulate the polarization of microglia by Sirt1 agonist or antagonist in vivo and in vivo respectively.

Shh, Ptc-1, Smo and Gli-1 are principle components of the canonical Shh signaling pathway, which is maintained in an ‘off’ state in the absence of Shh ligand for Ptc and inhibition of Smo [21]. When Shh binds to the Ptc-1 receptor, inhibition of Smo is relieved, thereby activating Gli transcription factors and allowing Gli proteins to translocate from the cytoplasm into the nucleus, further mediating the downstream effects of the Shh signaling pathway [41]. In the 1-methyl-4-phenyl-1,2,3,6-tetrahydropyridine (MPTP)-induced in vivo model of Parkinson’s disease, activated microglia were found to express Shh, and most neural cells, except oligodendrocytes, responded to microglia-derived Shh in MPTP-treated substantia nigra [42]. Moreover, activation of Shh signaling by purmorphamine inhibited activation of lipopolysaccharide-treated BV2 microglia through the PI3K/AKT pathway and protected dopaminergic neurons in a mouse model of Parkinson’s disease [43]. Lai et al. [44] reported that epigallocatechin gallate and minocycline increased the expression of Shh and that the anti-inflammatory effects of brain-derived neurotrophic factor were mediated by erythropoietin/Shh in microglia. These previous studies indicated that Shh/Gli-1 signaling may represent a potential therapeutic target for the regulation of microglial activation in central nervous system diseases. Furthermore, the findings of the present study were consistent with other researches which demonstrated that inhibition of microglial overactivation was closely associated with the Shh signaling pathway. Therefore, identifying drugs or targets activating Shh signaling pathway may exert neuroprotective effects by inhibiting microglial activation and reducing inflammation following OGD/R injury or cerebral ischemic injury.

However, there remain several questions to be further elucidated. For example, whether pretreatment with resveratrol, SRT1720 and sirtinol can affect the viability, apoptosis rate, activation and neuroinflammation of microglia, as well as the expression of Shh signaling-related molecules, prior to OGD/R injury in vitro and cerebral ischemic injury in vivo; whether Sirt1 can act through the Shh signaling pathway in microglia following cerebral ischemic injury in vivo and whether its effects are neuroprotective or neurorestorative; and whether Sirt1 can promote the polarization of microglia towards the ‘M1’ or ‘M2’ subtype. Is ‘M1’ microglia polarized into ‘M2’ subtype or is ‘M2’ microglia polarized into ‘M1’ subtype when administered following OGD/R injury in vitro or cerebral ischemic injury in vivo, and whether it is involved in the Shh signaling pathway. In the future, these issues will be further investigated by conducting animal experiments in vivo.

## Conclusion

To sum up, the findings of the present study may provide insight into the mechanism underlying Sirt1-regulated activation of microglia and neurological inflammation in the acute phase of stroke (Fig. 3). In the future, it will be necessary to examine how Sirt1 regulates the Shh/Gli-1 signaling pathway and to confirm the results of the present study in vivo.

## Supplementary Information

Below is the link to the electronic supplementary material.

Supplementary material 1 (TIF 342.0 kb)

## Data Availability

The datasets used and/or analyzed during the present study may be obtained from the corresponding author on reasonable request.
