# Supplementary figures and images for: Sirt1 regulates microglial activation and inflammation following oxygen-glucose deprivation/reoxygenation injury by targeting the Shh/Gli-1 signaling pathway
Source: Mol Biol Rep. 2023 Feb 1;50(4):3317–27. doi: 10.1007/s11033-022-08167-6 (PMC10042964; doi:10.1007/s11033-022-08167-6)

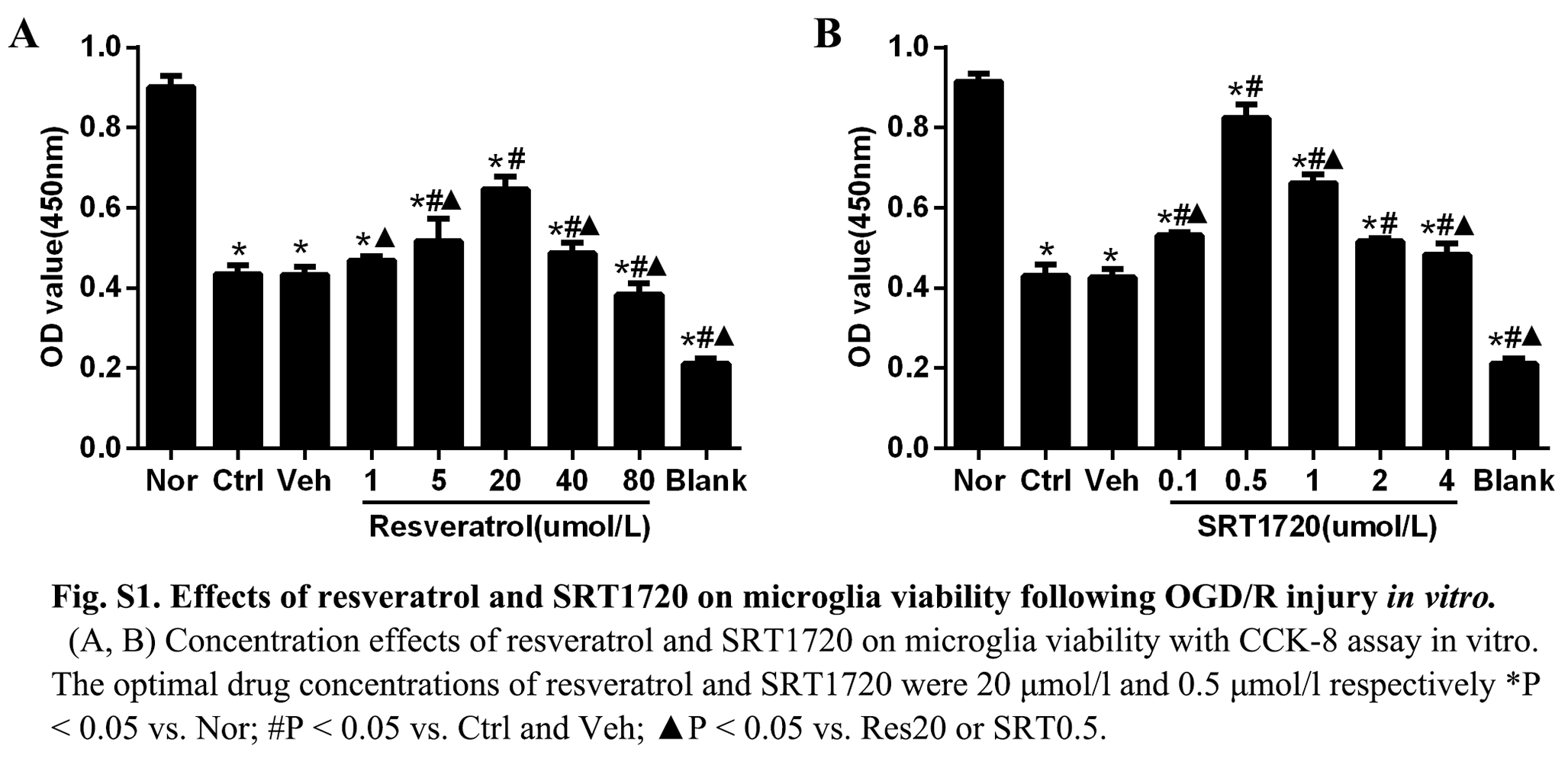

Supplement: Supplementary file 1 — Supplementary material 1 (TIF 342.0 kb) [file 11033_2022_8167_MOESM1_ESM.tif]
